# Editorial: Biofilms from a Food Microbiology Perspective: Structures, Functions, and Control Strategies

**DOI:** 10.3389/fmicb.2016.01938

**Published:** 2016-11-30

**Authors:** Avelino Álvarez-Ordóñez, Romain Briandet

**Affiliations:** ^1^Department of Food Hygiene and Technology and Institute of Food Science and Technology, University of LeónLeón, Spain; ^2^Micalis Institute, INRA, AgroParisTech, Université Paris-SaclayJouy-en-Josas, France

**Keywords:** biofilms, structure, control, food safety, food quality, bioprotection

Materials and equipment in food processing industries are colonized by surface-associated microbial communities called biofilms. In these biostructures microorganisms are embedded in a complex organic matrix composed essentially of polysaccharides, nucleic acids, and proteins. This organic shield contributes to the mechanical biofilm cohesion and triggers tolerance to environmental stresses such as dehydration or nutrient deprivation. Notably, cells within a biofilm are more tolerant to sanitation processes and the action of antimicrobial agents than their free living (or planktonic) counterparts. Such properties make conventional cleaning and disinfection protocols normally not effective in eradicating these biocontaminants. Biofilms are thus a continuous source of persistent microorganisms, including spoilage and pathogenic microorganisms, leading to repeated contamination of processed food with important economic and safety impact. Alternatively, in some particular settings, biofilm formation by resident or technological microorganisms can be desirable, due to possible enhancement of food fermentations or as a means of bioprotection against the settlement of pathogenic microorganisms.

In the last decades substantial research efforts have been devoted to unraveling mechanisms of biofilm formation, deciphering biofilm architecture, and understanding microbial interactions within those ecosystems. However, biofilms present a high level of complexity and many aspects remain yet to be fully understood. A lot of attention has been also paid to the development of novel strategies for preventing or controlling biofilm formation in industrial settings. Further research needs to be focused on the identification of new biocides effective against biofilm-associated microorganisms, the development of control strategies based on the inhibition of cell-to-cell communication, and the potential use of bacteriocins, bacteriocin-producing bacteria, phage, and natural antimicrobials as anti-biofilm agents, among others.

This research topic aims to provide an avenue for dissemination of recent advances within the “biofilms” field, from novel knowledge on mechanisms of biofilm formation and biofilm architecture to novel strategies for biofilm control in food industrial settings.

The research topic comprises three review articles, one perspective and 11 original research articles. Most of the contributions cover the most recent investigations on aspects related to the structures, architecture, and strategies for the control of biofilms formed by pathogenic or spoilage microorganisms on food processing surfaces, while two contributions are focused on the evaluation of biofilm formation by resident, technologically important or desirable microorganisms.

Various contributions deal with biofilms formed by strains of *Bacillus* spp. The review article by Majed and co-authors discusses the state-of-the-art on biofilms produced by *Bacillus cereus*, and by the two closely related pathogens, *Bacillus thuringensis* and *Bacillus anthracis* (Majed et al.). The review summarizes economic issues caused by *B. cereus* biofilms, the ecological and functional impact of biofilms in their lifecycle and management strategies implemented to control them. The research article by Hayrapeytan and co-authors shows the existence of intraspecies variability in the genome-encoded repertoire of iron-transporting systems and in the ability to grow and form biofilms in the presence of complex iron sources within *B. cereus*, which may influence *B. cereus* survival and persistence in food-related niches (Hayrapetyan et al.). Duanis-Assaf and co-authors report in their research article that lactose may induce biofilm formation by *Bacillus subtilis* through a quorum sensing dependent (LuxS) pathway (Duanis-Assaf et al.). In particular, they demonstrate that lactose induces formation of biofilm bundles, an increase in autoinducer-2 production in response to lactose, and an up-regulation of two gene operons responsible for extracellular matrix synthesis (e.g. *eps* and *tapA*).

In relation to *Campylobacter jejuni* biofilms, Brown and co-authors show in their contribution that extracellular DNA (eDNA) is an important component of *C. jejuni* biofilms formed on stainless steel surfaces (Brown et al.). The authors also evidence that eDNA may also contribute to the spread of antimicrobial resistance in *C. jejuni*. Finally, they report that degradation of eDNA by DNase I leads to rapid biofilm detachment, which shows promise for the control of *C. jejuni* biofilms in food industries. The research article by Turonova and co-authors reports that acclimation of two *C. jejuni* strains to oxygen-enriched conditions leads to a significant enhancement of biofilm formation during the early stages of the process, indicating that oxygen demand for biofilm formation is higher than for planktonic growth (Turonova et al.). The authors also identify the regulator CosR as a key protein in the maturation of *C. jejuni* biofilms. The research article by Bronnec and co-authors is aimed at evaluating the adhesion capacity and the ability to develop a biofilm of *C. jejuni* Bf, an atypical clinical isolate able to survive and grow under aerobic conditions (Bronnec et al.). The authors show that *C. jejuni* Bf can adhere to abiotic surfaces and human epithelial cells and can develop biofilms under both microaerobiosis and aerobiosis. They also conclude, from whole genome sequencing and transcriptomic analyses, that the behavior of this strain under aerobic atmosphere may result from the combination of different insertions and mutations and the modification of regulatory processes.

Two contributions are related to biofilms formed by strains of *Staphylococcus* spp. The perspective article by Oniciuc and co-authors shows that protein-based matrices are of relevance for the architecture of biofilms produced by *Staphylococcus aureus* strains isolated from food samples, as opposed to studies existing in the literature mentioning the predominance of exopolysaccharide-based matrices in biofilms formed by clinical and environmental isolates (Oniciuc et al.). Fagerlund and co-authors describe in their research article that the biofilm matrix composition has a significant impact on the efficacy of cleaning and disinfection agents against food associated Staphylococci (Fagerlund et al.). The authors show that some strains of *Staphylococcus* spp., able to form biofilms with a polysaccharide matrix, are resistant to benzalkonium chloride disinfectants, which are on the contrary effective for the removal of biofilms with a proteinaceous matrix.

Regarding biofilms formed by the foodborne pathogen *Listeria monocytogenes*, Zetzmann and co-authors report in their contribution that biofilms of *L. monocytogenes* are DNase-sensitive at low ionic strength conditions, which might induce bacterial lysis and chromosomal DNA release (Zetzmann et al.). This suggests that DNase I treatment is an attractive option to prevent or remove *L. monocytogenes* biofilms in food processing environments, where low nutrient concentrations and increased osmotic pressures are frequently found conditions. Puga and co-authors evaluate by confocal laser scanning microscopy changes in spatial organization, biovolume, viable cell content and substratum surface coverage of biofilms produced on glass by *L. monocytogenes* in co-culture with *Pseudomonas fluorescens* (Puga et al.). The authors conclude: “when this dual-species consortium develop biofilms on a solid surface, species interactions, cold stress and aging contribute to a more compact structure than the one built by *P. fluorescens* in single species biofilms.”

Two review articles are related to strategies for the control of biofilms formed by pathogenic or spoilage microorganisms. Gutiérrez and co-authors examine environmental factors determining biofilm development in food processing equipment and discuss available information and future prospects on the use of bacteriophage-derived tools as successful disinfectants for the removal of biofilms (Gutiérrez et al.). On the other hand, Coughlan and co-authors discuss the problems associated with bacterial biofilms in the food industry and summarize recent strategies explored to inhibit biofilm formation, with special focus on those targeting quorum sensing (Coughlan et al.).

Two original research articles deal with biofilm formation by desirable microorganisms. The research article by Bastard and co-authors shows that *Oenococcus oeni* produces biofilms capable of efficient malolactic fermentation during winemaking and that *O. oeni* biofilms attached to oak can modulate wood-wine transfer of volatile aromatic compounds during wine fermentation and aging (Bastard et al.). Gómez and co-authors report in their contribution that probiotic strains can be good alternatives for the control of biofilm production by pathogenic bacteria in food-related environments (Gómez et al.). The authors evaluate the probiotic properties of several bacteriocinogenic and non-bacteriocinogenic lactic acid bacteria (LAB) and develop protective biofilms with some good probiotic candidates and test them for exclusion of *L. monocytogenes, Escherichia coli* O157:H7 and *Salmonella* Typhimurium, obtaining promising results, with more than 6-log reductions in viable counts being achieved with some of the LAB strains.

Finally, in the last contribution, Ostrov and co-authors develop a method (Cleaning-In-Place model system) to evaluate the effectiveness of cleaning agents in removal of biofilm derived spores from the surfaces of stainless steel in milking equipment in dairy farms (Ostrov et al.).

This editorial summarizes the articles published in this Research Topic, in the confidence that readers will find this information useful with the most recent research on microbial biofilms from a food microbiology perspective. We sincerely hope that this collection of papers will prompt further research and contribute to advance the knowledge on food-related biofilms and to develop novel or improved strategies of food safety and quality management.

## Author contributions

AÁ and RB designed and wrote the Editorial.

### Conflict of interest statement

The authors declare that the research was conducted in the absence of any commercial or financial relationships that could be construed as a potential conflict of interest.

